# Mechanical Properties, Corrosion Damage Evolution Laws, and Durability Deterioration Indicators of High-Performance Concrete Exposed to Saline Soil Environment for 8 Years

**DOI:** 10.3390/ma18030565

**Published:** 2025-01-26

**Authors:** Xiaoming Wang, Hongfa Yu, Yongshan Tan, Chengyou Wu, Peng Wu, Haiyan Ma, Zhigang Ding, Lianxin Liu

**Affiliations:** 1 School of Civil Engineering, Qinghai University, Xining 810016, China; xiaoming_wang111@163.com (X.W.); wuchengyou86@163.com (C.W.); wupeng8686@126.com (P.W.); qhdxllx@126.com (L.L.); 2 College of Civil Aviation, Nanjing University of Aeronautics and Astronautics, Nanjing 211106, China; dzg6737@163.com; 3 College of Civil Science and Engineering, Yangzhou University, Yangzhou 225127, China; ystan@nuaa.edu.cn

**Keywords:** high-performance concrete, corrosion damage, strength, corrosion resistance coefficient

## Abstract

Most of the current studies rely on simulated brine corrosion environments and lack long-term investigations into concrete corrosion damage evolution under actual corrosive conditions. In this paper, high-performance concrete (HPC) with various mix ratios is designed in the context of the Qinghai Salt Lake region in China, and the evolution of corrosion damage of HPC with different water–binder ratios (*W*/*B*) and different fly ash (FA) admixtures under long-term field exposure conditions is obtained by testing the ultrasonic velocity and strengths of the HPC in the field exposure of the HPC in the Qinghai Salt Lake region. The results show that the corrosion resistance of HPC is related to its water–binder ratio and mineral admixture type and dosage under the exposure of 8 years in Qinghai Salt Lake area. HPC with a fly ash dosage of 15–35% and silica fume dosage of 10% exhibits better corrosion resistance when the water–binder ratio (*W*/*B*) is between 0.24 and 0.38. The dependence relationship between the corrosion resistance coefficient of HPC and the relative dynamic elastic modulus (*E_rd_*) and 28 d standard maintenance strength was also established. The *E_rd_* of HPC with a corrosion resistance coefficient of 0.80 or above was 0.73–0.93, not 0.60, which provides an important experimental basis for determining the corrosion damage index of HPC in the high-saline brine environment of the salt lake.

## 1. Introduction

The salt lake and saline soil environments surrounding the Qinghai Salt Lake region of China are highly corrosive, owing to elevated concentrations of salt and alkali. Concrete used in this environment faces common durability issues, such as corrosion of brine, corrosion of saline soil, and the corrosion of reinforcing bars caused by chloride ion erosion. These problems are often compounded by multifactorial durability changes [1]. Currently, research conducted both domestically and internationally on concrete in corrosive salt lake environments primarily focuses on single-ion corrosion. Many studies have been conducted on composite-ion corrosion as well. However, most of these studies rely on simulated brine corrosion environments and lack long-term investigations into concrete corrosion damage evolution under actual corrosive conditions.

Tumidajski et al. [2] summarized research from relevant literature [3,4,5,6,7,8,9,10] and concluded that during the extraction of potassium from seawater or salt lake brine, concrete structures in contact with high concentrations of magnesium salt brine are subjected to several destructive effects, and the primary corrosion products of concrete are Mg(OH)_2_ (abbreviated as MH), magnesium chloride oxide [Mg_2_(OH)_3_Cl·4H_2_O], magnesium silicate hydrate (M-S-H), monosulfoaluminate (C_3_A·CaSO_4_·12H_2_O, abbreviated as AFm), Friedel’s salt (C_3_A·CaCl_2_·10H_2_O), and expansive calcium sulfoaluminate (C_3_A·3CaSO_4_·32H_2_O, abbreviated as AFt), with C_3_A·CaCl_2_·10H_2_O inevitably converting into AFt. Yu [11] further expanded on the chemical corrosion mechanism of concrete in the Qinghai Salt Lake region, where a high concentration of magnesium hydroxide–magnesium, chloride–calcium, chloroaluminate–gypsum composites accelerate corrosion. The conclusion was that physical factors, such as salt-enriched crystallization, carbonation of corrosion products, and volumetric changes due to temperature differences in the salt lake’s harsh climatic conditions, contribute to the acceleration of concrete corrosion. Physical effects such as salt crystallization, corrosion product carbonation, and volumetric expansion caused by temperature variations are believed to significantly speed up chemical corrosion in concrete.

Ju [12] found that partially replacing cement with reactive admixtures significantly improved concrete’s resistance to sulfate attack. Smarzewski et al. [13] investigated the effect of cinder and foundry waste sand on the resistance of high-performance concrete (HPC) to salt attack using the experimental method outlined in EN 12370:2001 [14] for resistance to salt crystallization. Harbec et al. [15] compared the effects of glass and silica fumes on HPC’s resistance to sulfate attacks and mortar’s resistance to sulfate erosion. Yu [16] found that high-strength, non-air-entraining HPC exhibited excellent resistance to corrosion due to saline soil and analyzed the optimization mechanism for improving the corrosion resistance of HPC.

Ma et al. [17] investigated the performance of ordinary Portland cement concrete (OPC) and HPC after exposure to wet and dry cycles in various salt lake brines, addressing the severe erosion of concrete in salt lake regions. Ding et al. [18] investigated the corrosion of HPC exposed to high-concentration Na^+^-Cl^−^-SO_4_^2−^ brine with subsurface salinity levels of 180 g/L in the Xining Basin. They found that HPC performed significantly better than OPC in freeze–thaw resistance. The investigation into brine corrosion revealed that the relative dynamic modulus of elasticity (*E_rd_*) of both plain concrete and reinforced concrete beams remained above 90%. Additionally, the compressive corrosion coefficient (Kc) of plain concrete exceeded 0.8 after 12 years of exposure to corrosive conditions, with plain concrete under an initial stress ratio of 0.30 and reinforced concrete beams under a stress ratio of 0.70 in high-concentration brine environments. Even with a protective layer thickness of only 15 mm, the concrete in reinforced concrete beams provides effective protection for the steel reinforcement. Li et al. [19] investigated the long-term mechanical behavior of HPC specimens in the brine environment of an underground salt lake in Xining, with a salt content of 180,894.5 mg/L. The results showed that as the exposure time to salt lake brine increased, the compressive and tensile strength of HPC, along with the relative dynamic modulus of elasticity, initially increased and then decreased. After 10 years of exposure to salt lake brine, the compressive strength of the HPC specimens remained significantly higher than their original strength, and corrosion damage to the concrete was minimized. Wang et al. [20] studied the effects of the coupling of corrosion and the alkali–silica reaction (ASR) in salt lake brine on the mechanical properties of HPC doped with ASR inhibitors. They found that as the equivalent alkali content in HPC increased, corrosion damage worsened, leading to a reduction in tensile strength at break.

This study aimed to determine the relative dynamic elastic modulus (*E_rd_*), various strengths, and corrosion resistance coefficients after 2920 days of exposure to the saline soil environment at Qarhan Salt Lake. It investigated the evolution of corrosion damage in HPC with varying water–cement ratios and different fly ash admixtures under long-term field exposure in the Qinghai Salt Lake region. The objective was to provide insights into the engineering application of concrete in high-altitude areas of the Tibetan Plateau, where high concentrations of saline soil are present.

## 2. Materials and Methods

### 2.1. Raw Materials

The cement used in this study is ordinary Portland cement, grade 42.5, manufactured by Qinghai Datong Yuanshuo Cement Co., Ltd. (Xining, China). The fly ash (FA) is Class I, sourced from the Datong Qiaotou Power Plant. (Xining, China). and the silica fume (SF) is produced by the Qinghai Minhe Magnesium Plant. (Xining, China). The fineness of fly ash is 11.5%, the specific surface area is 356 m^2^/kg, the water requirement ratio is 95%, the density is 2.1 g/cm^3^, the and bead content is 92.5%; the specific surface area of silica fume is 18 m^2^/g, the water content is 0.66%, the activity of volcanic ash is 115.5%, the average particle size is 0.1–0.2 μm, the stacking density is 280 kg/m^3^, and the density is 2.2 g/cm^3^. The main performance indexes of cement are shown in Table 1, and the chemical composition of cementitious material is shown in Table 2.

The sand used is natural sand from the Beichuan River, in Xining City, with a fineness modulus of 3.0, a mud content of 2.9%, an apparent density of 2670 kg/m^3^, and a bulk density of 1720 kg/m^3^. The aggregate used is artificially crushed stone from the Beichuan area of Xining City, featuring a continuous gradation of 5–20 mm, maximum particle size of 20 mm, crushing index of 8.06%, mud content of 0.6%, apparent density of 2870 kg/m^3^, and bulk density of 1330 kg/m^3^. 

The admixtures used include the fixed dialer number (FDN) high-efficiency water-reducing agent and the HAJ-2 high-efficiency air-entraining and water-reducing agent, both manufactured by the Beijing Muhu Admixtures Factory (Beijing, China). The FDN agent, in liquid form, is primarily composed of naphthalene sulfonate formaldehyde high condensate, with a solid content of 33%. The recommended dosage is 1.5%, and the water reduction rate exceeds 25%. Additionally, it contains K_2_O at 0.000562% and Na_2_O at 0.427518%. The HAJ-2 agent, in solid granule form, is primarily made of naphthalene sulfonate formaldehyde condensate and sulfonated melamine formaldehyde condensate, offering a water reduction rate of 10% or more.

### 2.2. Concrete Mixing Ratio

In this experiment, nine concrete mix proportions were designed. For HPC with FA, mix designs ranged from C20 to C40. For HPC with both FA and SF, the range extended from C30 to C70. Specifically, C20 to C40 concrete included single additions of 23%, 33%, and 43% FA, while C30 to C70 concrete utilized a dual addition of SF and FA, with SF content at 10% and FA content at 15%, 25%, and 35%, respectively.

**Figure 1:**
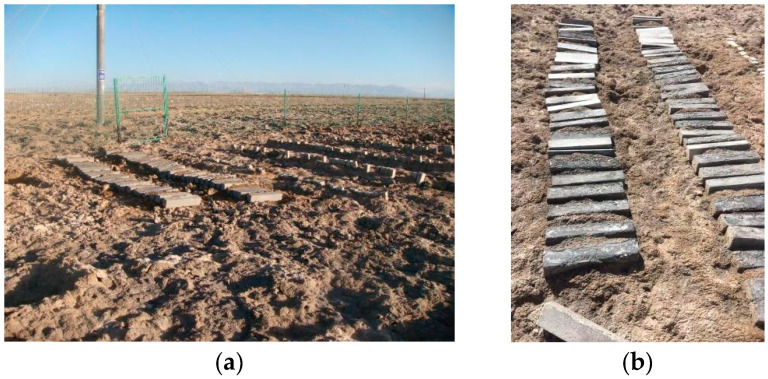
The High-Performance Concrete Saline Soil Exposure Station (Golmud City, Qinghai Province, China). (**a**) On site—taken in 2015; (**b**) specimen—taken in 2022.

The specimen dimensions were 100 mm × 100 mm × 515 mm, with a copper measuring head embedded at each end. The series of concrete specimens were produced by our research group in 2003. After casting, they were cured for 24 h under standard conditions, de-molded, and subsequently numbered. The concrete specimens were cured in a simulated dry cold (DC) and dry hot (DH) environmental laboratory. The mix proportions of the concrete specimens are detailed in Table 3 and Table 4, while the 28 d cube compressive strength under different curing conditions is presented in Table 5 and Table 6. After five years of curing, the specimens were placed in an outdoor exposure environment in Xining City, Qinghai Province. In July 2015, a portion of these specimens was transported to the HPC Saline Soil exposure station at Qarhan Salt Lake (Figure 1), Golmud City, Qinghai Province, China, for further exposure. Testing was then conducted in 2023 at the Civil Engineering College Laboratory, Qinghai University, with the outdoor exposed specimens in Xining City serving as the control group.

According to the meteorological data of the Qinghai–Tibet Plateau, the Qarhan Salt Lake exposure station experiences a DC climate throughout the year, with predominantly DC winters and DH summers. The DH environmental simulation laboratory maintains a temperature of (35 ± 2) °C with a relative humidity of (40 ± 5)%, while the DC laboratory is set at (5 ± 2) °C with a relative humidity of (40 ± 5)%.

### 2.3. Saline Soil Exposure Station

**Figure 2:**
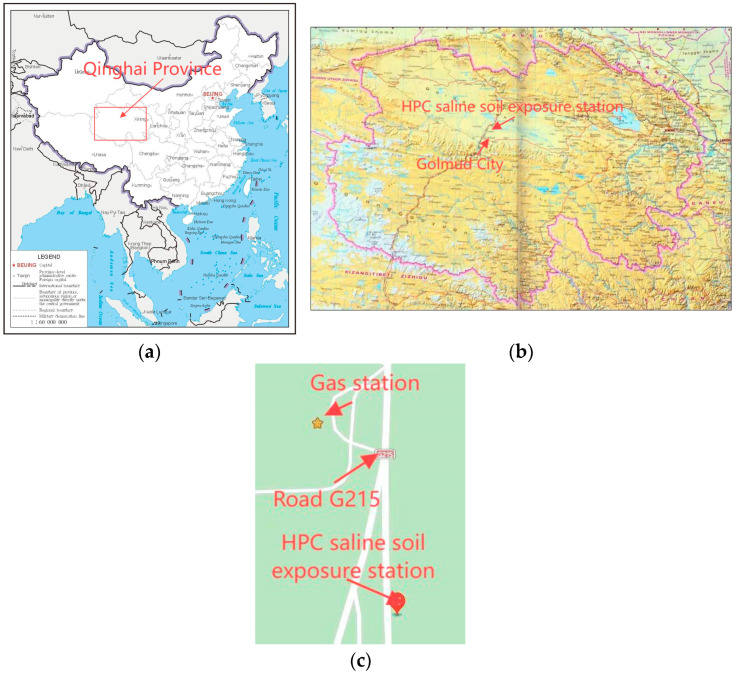
Qarhan Salt Lake and the specific location of the saline soil exposure station. (**a**) Qinghai Province, China; (**b**) Golmud City, Qinghai Province; (**c**) location of the HPC saline soil exposure station.

The saline land surrounding the salt lake is extensive (Figure 2), with some soils containing fine crystal salt grains and others containing salt blocks measuring 5 to 8 mm. The saline soil has a high concentration of soluble salts and is classified into three zones based on the degree of salinization: light saline soil, strong saline soil, and extremely saline soil. This classification primarily focuses on chloride-based saline soils. Light saline soil has a salt content between 0.4% and 4%, strong saline soil has a salt content between 3% and 13%, and extremely saline soil has a salt content ranging from 5% to 44%. Using the Qarhan Salt Lake in Qinghai as an example, there is approximately 30 km of light saline soil, followed by 16.5 km of strong saline soil, and about 5 km of extremely saline soil between Golmud and Qarhan.

Table 7 lists the chemical composition of the surface saline soil in the Qarhan Salt Lake. The results indicate that the saline components are similar to brine, with the highest salt content reaching 43.66%. Even in areas with light saline soil, the salt content of the surface soil remains relatively high. The HPC exposure station in the salt lake (Figure 1) is located in a strong saline soil zone, where the chemical composition of the soluble salts in the soil is as listed in Table 7. The salt content is 37.17%, and the concentrations of ions such as Mg^2+^, Cl^−^, SO_4_^2−^, and CO_3_^2−^ are 0.5%, 17.88%, 0.7%, and 4.31%, respectively.

**Figure 3:**
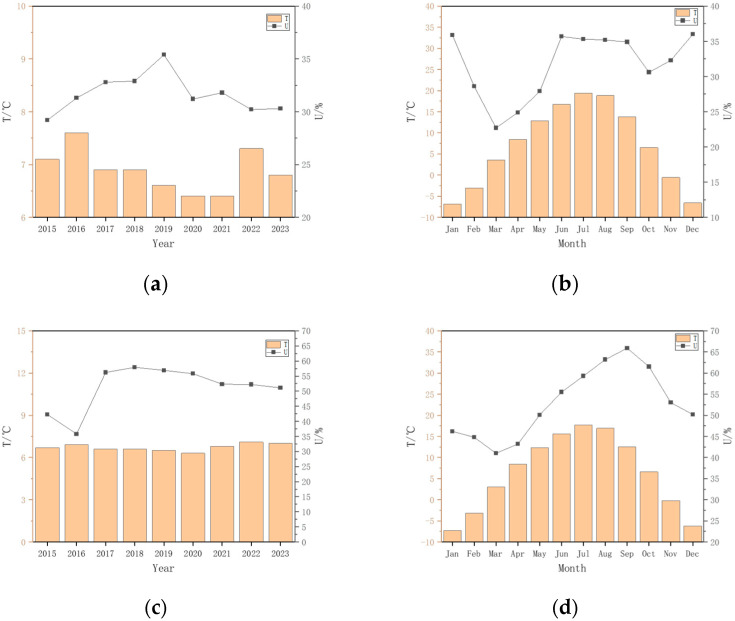
Atmospheric Environmental Temperature and Humidity Changes in the Qarhan Salt Lake Area and Xining Area of Qinghai. (**a**) Average annual T and U in the Qarhan Salt Lake area; (**b**) average monthly temperature and precipitation in the Qarhan Salt Lake area; (**c**) average annual T and U in the Xining area; (**d**) average monthly T and U in the Xining area.

The temperature (T) and relative humidity (U) changes in the atmospheric environment of the Qarhan Salt Lake area and the Xining area from 2015 to 2023 are shown in Figure 3.

### 2.4. Test Methods

#### 2.4.1. Test Flow

(1) By determining the ultrasonic velocity inside the chronically exposed concrete specimens in the Salt Lake region, relative dynamic elastic modulus, corrosion resistance coefficient, and corrosion damage were obtained;
(2) By determining the macroscopic mechanical properties of long-term exposed concrete specimens in the salt lake area, compressive strength, flexural strength, split tensile strength, and prismatic axial compressive strength were obtained;
(3) The above data are synthesized to explore the effect of mix ratio on mechanical strength, the interrelationship between mechanical strengths, the corrosion damage law of concrete specimens, and the evolution law between mechanical strength and corrosion damage.

#### 2.4.2. Strength Test Method

**Figure 4:**
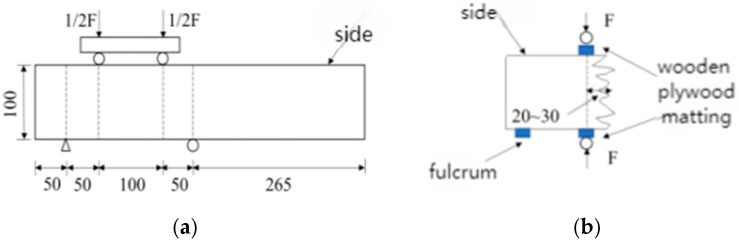
Schematic diagram of concrete strength test. (**a**) Flexural Strength Test. (**b**) Splitting tensile Strength Test.

This experiment utilized the YAW 4306 microcomputer-controlled electro-hydraulic servo pressure testing machine manufactured by Meters Industrial Systems (Xining, China) Co. The testing machine has a capacity of 3000 kN, maximum platen distance of 400 mm, and measurement range of 2%-FS. According to the relevant provisions of the “Standard for Methods of Examining the Physical and Mechanical Properties of Concrete”, the prismatic flexural strength and cubic splitting tensile strength of concrete specimens were measured, as shown in Figure 4.

**Figure 5:**
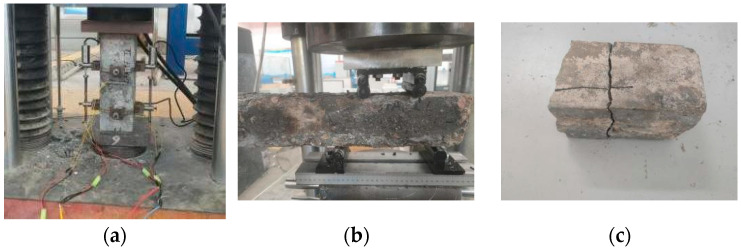
Concrete Strength Test Chart. (**a**) Axial compressive strength test. (**b**) Flexural Strength Test. (**c**) Splitting tensile failure mode.

For the prismatic cylinder uniaxial compression test, the remaining portion of the test specimen used for the flexural strength test was cut into dimensions of 100 mm × 100 mm × 300 mm according to the specifications. Care was taken to ensure that both ends of the test specimen were flat, smooth, and parallel to avoid eccentric damage during the uniaxial test, which could affect the accuracy of the results. The field test setup is illustrated in Figure 5.

(1) Axial compressive strength

According to the “Standard for Inspection Methods of Physical and Mechanical Property of Concrete”, the axial compressive strength of the HPC specimen is calculated using Equation (1):

(1) $$f_{c}=\frac{F}{A}$$

where *f_c_* is the axial compressive strength of concrete (MPa), *F* is the specimen destructive load (N), and *A* is the concrete specimen compressive area (mm^2^). The axial compressive strength of prismatic specimens measured using non-standard specimens (100 mm × 100 mm × 300 mm) are multiplied by a size conversion factor of 0.95.

(2) Flexural strength

According to the “Standard for Inspection Methods of Physical and Mechanical Property of Concrete”, the flexural strength of HPC specimen is calculated using Equation (2):

(2) $$f_{f}=\frac{Fl}{bh^{2}}$$

where *f_f_* is the flexural strength of concrete (MPa), *F* is the specimen destructive load (N), *l* is the span between the supports (mm), *b* is the specimen cross-section width (mm), and h is the specimen cross-section height (mm). As the span of the flexural strength test specimen is 200 mm, the conversion factor is calculated to be 0.75; therefore, the flexural strength results must be converted to non-standard specimen size and span, i.e., multiplied by the conversion factors of 0.85 and 0.75.

(3) Splitting tensile strength

According to the “Standard for Inspection Methods of Physical and Mechanical Property of Concrete”, the splitting tensile strength of HPC specimen is calculated using Equation (3):

(3) $$f_{sp}=\frac{2f}{\pi A}=0.637\frac{F}{A}$$

where *f_sp_* is the concrete splitting tensile strength (MPa), *F* is the specimen destructive load (N), and *A* is the specimen splitting surface area (mm^2^). In addition, the splitting tensile strengths measured using non-standard specimens (100 mm × 100 mm × 100 mm) were multiplied by a size conversion factor of 0.85.

#### 2.4.3. Corrosion Damage Testing (Ultrasonic Countermeasure)

**Figure 6:**
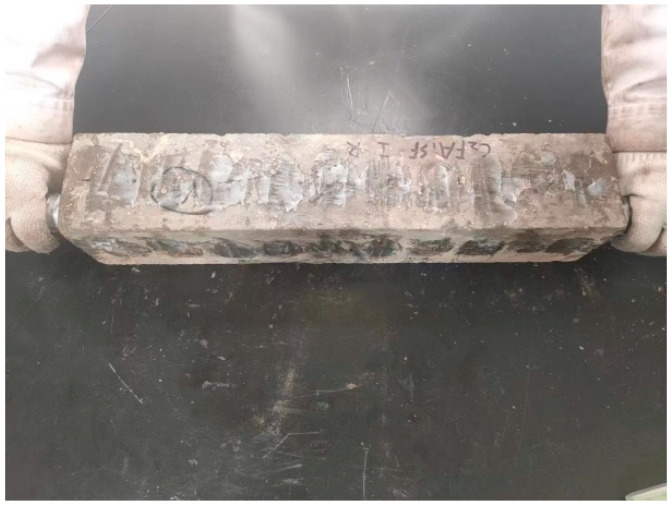
Ultrasonic Velocity Measurement.

The corrosion damage testing was conducted using a non-metallic ultrasonic testing analyzer (ZT803) produced by Beijing Zhongtuo Science and Technology Co., Ltd. (Beijing, China). The test followed the “Ultrasonic Method for Detecting Defects in Concrete Technical Regulations” to determine the ultrasonic velocity in the concrete. The “pair of test method” was employed, where two transducers were placed on opposite sides of the 100 mm × 100 mm specimen, along the length, to measure the ultrasonic velocity, as shown in Figure 6.

Ultrasonic pulse velocity testing is a widely applied non-destructive method to determine the elastic properties of concrete. Assuming the damage to the concrete is isotropic and the Poisson’s ratio remains unchanged, the relative dynamic elastic modulus of concrete is calculated using Equation (4):

(4) $$E_{rd}=\frac{E_{n}}{E_{0}}=\frac{v_{n}^{2}}{v_{0}^{2}}=\frac{t_{0}^{2}}{t_{n}^{2}}\times 100\%$$

where *E_rd_* is the relative kinetic elastic modulus of concrete (%); *E_n_*, *v_n_*, and *t_n_* are the kinetic elastic modulus of concrete at different ages (N·mm^−2^), speed of sound (m·s^−1^), and acoustic time (μs), respectively; *E*_0_, *v*_0_, and *t*_0_ are the kinetic elastic modulus of concrete before the exposure curing (N·mm^−2^), acoustic speed (m·s^−1^), and acoustic time (μs), respectively.

Corrosion damage (*D*) is calculated using Equation (5):

(5) $$D=1-E_{rd}$$

#### 2.4.4. Anti-Corrosion Coefficient

The corrosion resistance coefficient (*K*) directly reflects the strength loss of corroded concrete. In this study, the corrosion resistance coefficient, *K*, is calculated using Equation (6):

(6) $$K=\frac{f_{c}}{f_{c0}}$$

where *f_c_*_0_ is the axial compressive strength (MPa) of concrete not attacked by the salt lake and after 8 years of natural exposure in Xining area, and *f_c_* is the axial compressive strength (MPa) of concrete after 8 years of on-site exposure and corrosion in the salt lake area.

#### 2.4.5. HPC Corrosion Product XRD Test

The surface mortar sample is taken from a depth of 3–5 mm on the surface of the specimen, and the core mortar sample is taken from the center of the specimen. After primary grinding, sieving, and secondary sieving, specimens meeting the fineness requirement of 200 mesh were selected. An X-ray diffractometer (D/max-2500PC, CuKα target, Rigaku Corporation, Tokyo, Japan) was employed to conduct XRD phase analysis of the corrosion products. The technical parameters of X-ray diffractometer are as follows: a voltage of 30 kV, a current of 30 mA, a scanning speed of 4 °/min, and a scan range of 2θ = 5~70°.

#### 2.4.6. SEM Test of the HPC Microstructure

The corrosion specimens of HPC were subjected to vacuum gold spraying treatment using the ETD-2000 miniature ion sputtering instrument produced by Beiing Boyuan Micro Nano Technology Co., Ltd. (Beijing, China) to terminate the corrosion reaction. The technical parameters of the vacuum gold spraying treatment were as follows: a gold spraying current of 10 mA, an air pressure of 0.2 mbar (20 Pa), and a gold spraying thickness of 20 nm. Using the method for preparing XRD samples, specimens mainly composed of cement slurry were prepared for SEM testing to observe the microstructure of the specimens. SEM testing was performed using the COXEM EM-30PLUS desktop scanning electron microscope (SEM) from Silicon Valley Daejeon City of the Republic of Korea, with parameters including a resolution of less than 5 nm, an acceleration voltage of 20 kV, and a current of 15 mA.

## 3. Results and Analysis

### 3.1. Surface Corrosion State of HPC After 8 Years of Field Exposure in the Salt Lake Region

**Figure 7:**
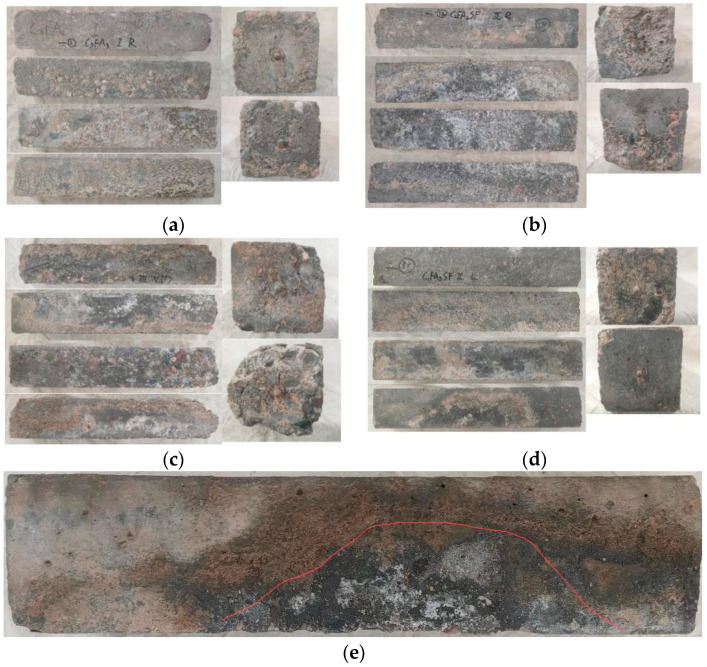
Appearance morphology of HPC specimens exposed for 8 years in the Qinghai salt lake area. (**a**) DH-C5FA2. (**b**) DH-C3FA2SF. (**c**) DC-C6FA2. (**d**) DC-C1FA3SF. (**e**) Peeling area of arched cement mortar.

Figure 7 shows the appearance and morphology of HPC specimens exposed for 8 years in the Salt Lake area of Qinghai. The four 100 mm × 515 mm-long sections on the left side of the figure represent the top surface, side 1, bottom surface, and side 2, respectively. The two 100 mm × 100 mm sections on the right side show the left and right end surfaces.

As seen in Figure 7, most of the specimens exposed to the salt lake area for 8 years showed severe corrosion. Many specimens exhibited surface pits, significant peeling, and missing corners or cracks. Only a small number of specimens, such as the one shown in Figure 7d, remained relatively intact. Based on the visual inspection, it is evident that the HPC specimens exposed to the salt lake environment for 8 years experienced serious corrosive damage.

The bottom surface of the HPC specimens (buried) was severely corroded, with the surface cement mortar almost completely eroded, exposing the aggregate. The top surface of the majority of the HPC specimens (exposed) was significantly affected by weathering because of the dry, cold, and hot climates, resulting in numerous potholes. On the sides of the HPC specimens (semi-buried), the corrosion was more severe closer to the ground, where the cement mortar layer was more eroded. In contrast, the not buried parts of the cement mortar layer remained relatively intact. Notably, on the semi-buried sides of the HPC specimens, arch-shaped peeling areas were observed, indicating that the closer the section was to the ground, the more severe the corrosion of the cement mortar layer became.

### 3.2. Strength Patterns of Concrete After 8 Years of Field Exposure in the Salt Lake Region

#### 3.2.1. Axial Compressive Strength and Its Influencing Factors

##### Effect of Water–Binder Ratio on Axial Compressive Strength

**Figure 8:**
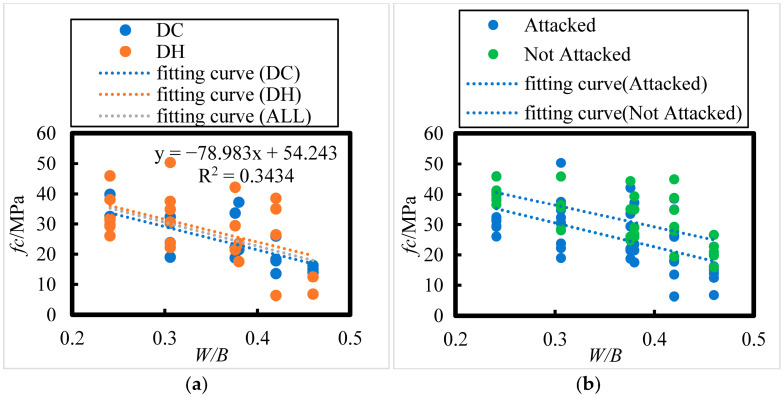
Effect of Water–Binder Ratio on the Axial Compressive Strength of HPC under Dry Cold, Dry Heat, Attacked, and Not attacked Conditions. (**a**) DH, DC, ALL. (**b**) Attacked, Not Attacked.

The effect of water–binder ratio on the axial compressive strength of concrete under 8 years of field exposure in the Salt Lake region is shown in Figure 8; the following relationship between axial compressive strength (*f_c_*) and water–binder ratio (*W*/*B*) can be observed:

(7) $$f_{c}=-75.19\;W/B+54.1\;\;\;\;\;\mathrm{DH},\;R\;=\;0.50$$

(8) $$f_{c}=-77.027\;W/B+52.224\;\;\mathrm{DC},\;R\;=\;0.70$$

(9) $$f_{c}=-78.983\;W/B+54.243\;\;\mathrm{ALL},\;R\;=\;0.59$$

The number of HPC specimens cured in the DH environment was 23. According to the correlation coefficient test table (Table 8), the critical correlation coefficient at the 0.05 significance level for n = 23 is 0.396. For HPC specimens cured in the DC environment, the sample size is 20, and for all HPC specimens combined, the sample size is 43. The critical correlation coefficients at the 0.001 significance level for n = 20 and n = 43 are 0.652 and 0.49, respectively. All related correlation coefficients passed the test, confirming an extremely significant linear relationship between the axial compressive strength of the concrete and the water–binder ratio. Specifically, the axial compressive strength decreases as the water–binder ratio increases.

The initial curing environments (DC and DH) did not have a significant effect on the axial compressive strength of the concrete. As shown in Figure 8a, the axial compressive strength of the concrete ranges between 26.1 and 45.9 MPa, 23.1 and 50.3 MPa, and 18.7 and 42.1 MPa for water–binder ratios of 0.241, 0.306, and 0.376, respectively. For water–binder ratios of 0.38, 0.42, and 0.46, the compressive strength ranges from 17.5 to 37.2 MPa, 6.3 to 38.5 MPa, and 6.7 to 16.2 MPa, respectively.

As seen in Figure 8b, after 8 years of exposure in the Salt Lake region, the axial compressive strength of all concrete specimens decreased compared to that of the non-corroded specimens. The reduction in compressive strength for concrete with water–binder ratios of 0.241, 0.306, 0.376, 0.38, 0.42, and 0.46 was 13.32%, 16.40%, 20.74%, 21.03%, 24.27%, and 28.26%, respectively. This indicates that the higher the water–binder ratio, the greater the reduction in axial compressive strength.

##### Effect of Fly Ash Content on Axial Compressive Strength

**Figure 9:**
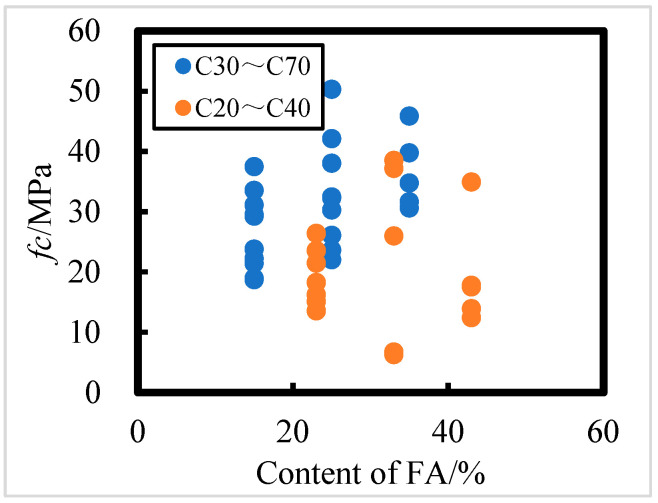
Effect of fly ash content on axial compressive strength of C20 to C40 and C30 to C70 HPC.

The effect of FA content on the axial compressive strength of concrete exposed in the field for 8 years in the Salt Lake area is shown in Figure 9; it is evident that the relationship between FA dosage and axial compressive strength of concrete is not significant, i.e., the content of FA has an effect on the axial compressive strength of concrete, but it is not the main factor. For C30 to C70 concrete, the higher the FA addition, the higher the axial compressive strength; for C20 to C40 concrete, the FA addition does not have a significant effect on its strength, and the axial compressive strength of C20 to C40 concrete fluctuates greatly when the FA content is 33% and 43%. It can be seen that HPC with a water–binder ratio of 0.241 and FA content of 35% has the strongest corrosion resistance according to axial compressive strength.

#### 3.2.2. Flexural Strength and Its Influencing Factors

##### Effect of Water–Binder Ratio on Flexural Strength

**Figure 10:**
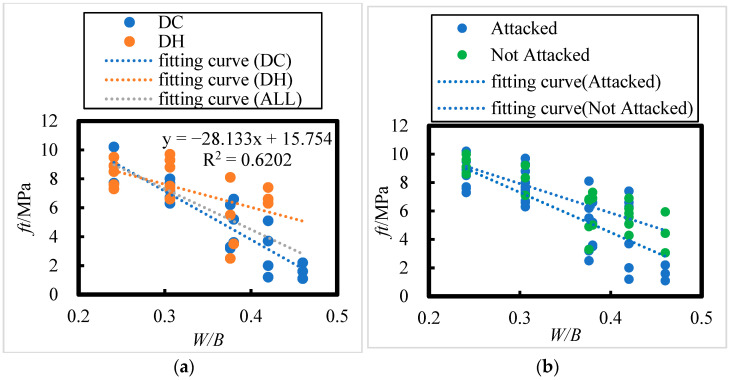
The effect of Water–Binder Ratio on the Flexural Strength of HPC under Dry Cold, Dry Heat, Attacked, and Not attacked Conditions. (**a**) DH, DC, ALL. (**b**) Attacked, Not Attacked.

The effect of water–binder ratio on the flexural strength of concrete under 8 years of field exposure in the Salt Lake region is shown in Figure 10; the relationship between flexural strength (*f_t_*) and water–binder ratio (*W*/*B*) can be summarized as follows:

(10) $$f_{t}=-15.945\;W/B+12.414\;\;\;\mathrm{DH},\;R\;=\;0.56$$

(11) $$f_{t}=-33.52\;W/B+17.21\;\;\;\;\;\;\mathrm{DC},\;R\;=\;0.90$$

(12) $$f_{t}=-28.133\;W/B+15.754\;\;\;\;\mathrm{ALL},\;R\;=\;0.79$$

The number of HPC specimens with initial curing environments of DH and DC are 19 and 21, respectively. From Table 8, the critical correlation coefficients at a significance level of 0.01 for n = 19 and n = 21 are 0.549 and 0.526, respectively. For the total number of HPC specimens (40), from Table 8, the critical correlation coefficient at a significance level of 0.001 for n = 40 is 0.49; all the related correlation coefficients passed the correlation coefficient test. This implies that there exists an extremely significant linear relationship between the flexural strength of concrete and the water–binder ratio. After 8 years of exposure in the salt lake area, the flexural strength of concrete decreases with an increase in the water–binder ratio. From Figure 10a, for water–binder ratios of 0.241, 0.306, and 0.376, the flexural strength of concrete is between 7.3 and 10.2 MPa, 6.3 and 9.7 MPa, and 2.5 and 8.1 MPa, respectively; when the water–binder ratios are 0.38, 0.42, and 0.46, the flexural strength of concrete is between 3.6 and 6.6 MPa, 1.2 and 7.4 MPa, and 1.1 and 2.2 MPa, respectively. From Figure 10b, it is evident that after 8 years of exposure in the salt lake area, the flexural strength of the concrete has decreased compared to that of the control concrete. The flexural strength of concrete with water–binder ratios of 0.241, 0.306, 0.376, 0.38, 0.42, and 0.46 decreased by 1.89%, 8.50%, 18.71%, 19.44%, 27.89%, and 39.35%, respectively. Therefore, the higher the water–binder ratio, the greater the rate of decrease in flexural strength.

##### Effect of Fly Ash Content on Flexural Strength

**Figure 11:**
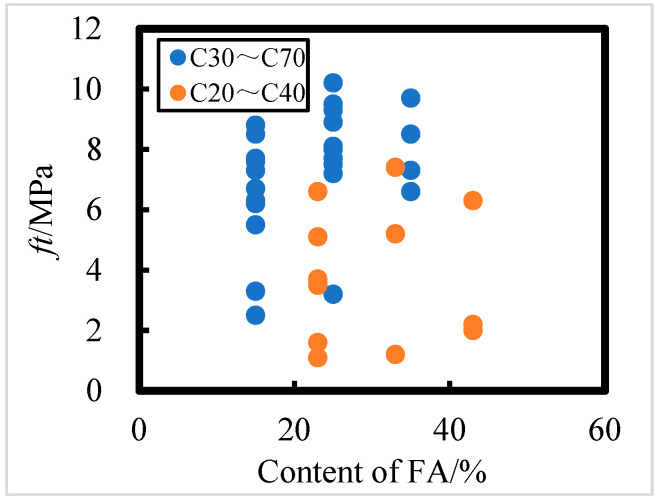
Effect of fly ash content on flexural strength of C20 to C40 and C30 to C70 HPC.

The effect of FA content on the flexural strength of concrete exposed in the field for 8 years in the Salt Lake region is shown in Figure 11; the effect of FA content on the flexural strength of concrete is not significant, i.e., although FA content does have an effect on the axial compressive strength of concrete, it is not a major factor. For C30 to C70 concrete, the higher the FA content, the greater the concrete flexural strength; for C20 to C40 concrete, the FA content has minimal effect on flexural strength. HPC with a water–binder ratio of 0.241 and a FA admixture of 35% has the strongest corrosion resistance according to the flexural strength.

#### 3.2.3. Splitting Tensile Strength and Its Influencing Factors

##### Effect of Water–Binder Ratio on Splitting Tensile Strength

**Figure 12:**
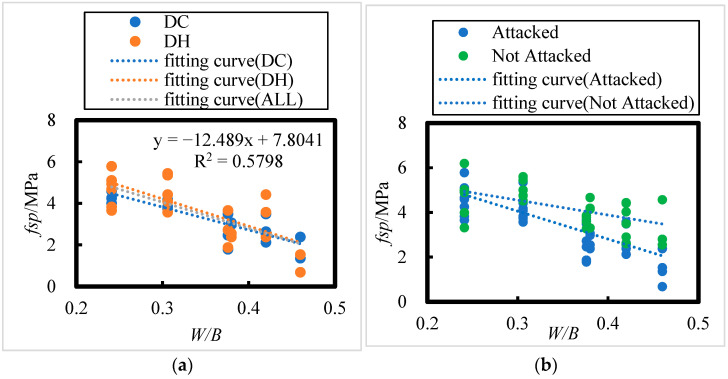
Effect of Fly Ash Content on Splitting Tensile Strength of HPC under Dry Cold and Dry Heat Conditions. (**a**) DH, DC, ALL; (**b**) Attacked, Not Attacked.

The effect of the water–binder ratio on the splitting tensile strength of concrete after 8 years of field exposure in the Salt Lake region is shown in Figure 12; the following relationship between splitting tensile strength (*f_sp_*) and water–binder ratio (*W*/*B*) can be obtained:

(13) $$f_{\mathrm{sp}}=-13.119\;W/B+8.1498\;\;\mathrm{DR},\;R\;=\;0.73$$

(14) $$f_{\mathrm{sp}}=-11.031\;W/B+7.1321\;\;\mathrm{DC},\;R\;=\;0.84$$

(15) $$f_{\mathrm{sp}}=-12.489\;W/B+7.8041\;\;\;\mathrm{ALL},\;R\;=\;0.76$$

The number of HPC specimen samples with initial curing environments of DH and DC is 23 and 20, respectively. According to the Table 8, the critical correlation coefficients at the 0.01 level of significance for n = 23 and n = 20 are 0.505 and 0.537, respectively. For all HPC samples combined (n = 43), the critical correlation coefficient at the 0.001 significance level is 0.474. All related correlation coefficients passed the correlation coefficient test.

It is evident that there is an extremely significant linear relationship between the splitting tensile strength of concrete and the water–binder ratio. After 8 years of exposure in the salt lake region, the splitting tensile strength of concrete decreases as the water–binder ratio increases. From Figure 12a, it can be observed that when the water–binder ratios are 0.241, 0.306, and 0.376, the splitting tensile strength ranges of the concrete are 3.8–5.8 MPa, 3.6–5.4 MPa, and 1.8–3.7 MPa, respectively. For water–binder ratios of 0.38, 0.42, and 0.46, the splitting tensile strength ranges are 2.4–3.1 MPa, 2.1–4.4 MPa, and 0.7–2.4 MPa, respectively.

From Figure 12b, it is evident that after 8 years of exposure in the salt lake region, the splitting tensile strength of the concrete decreased compared to that of non-corroded concrete. The reductions in splitting tensile strength for water–binder ratios of 0.241, 0.306, 0.376, 0.38, 0.42, and 0.46 were 3.04%, 11.71%, 23.13%, 23.86%, 31.76%, and 40.87%, respectively. These data show that the higher the water–binder ratio, the greater the rate of decrease in splitting tensile strength.

##### Effect of Fly Ash Content on Splitting Tensile Strength

**Figure 13:**
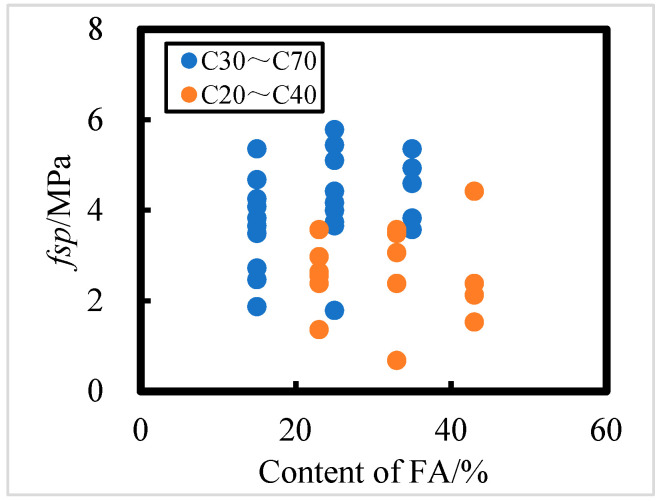
The effect of fly ash content on split tensile strength of C20 to C40 and C30 to C70 HPC.

The effect of the FA admixture on the splitting tensile strength of concrete exposed for 8 years in the Salt Lake region is shown in Figure 13. As seen in from Figure 13, the influence of fly ash on splitting tensile strength is not significant. For C30 to C70 concrete, higher fly ash content results in greater splitting tensile strength. However, for C20 to C40 concrete, the FA admixture has minimal impact on splitting tensile strength. Based on the data, HPC with a water–binder ratio of 0.241 and a FA admixture of 35% demonstrates the strongest corrosion resistance, as indicated by splitting tensile strength.

#### 3.2.4. Relationship Among Axial Compressive, Flexural, and Splitting Tensile Strengths of HPC After 8 Years of Exposure in the Salt Lake Region

##### Relationship Between Flexural and Axial Compressive Strengths

According to the “Standard for Test Methods of Mechanical Properties of Ordinary Concrete”, there is a conversion relationship between compressive and axial compressive strengths. For concrete below C50, the conversion factor is typically 0.76, while for concrete above C50, it ranges between 0.78 and 0.82 [21]. In this study, the conversion factor is taken as 0.76, meaning fc = 0.76 fcu.

Various organizations and departments, both domestic and international, have modeled the relationship between flexural and compressive strengths for different types of concrete. Notable models include those from the Japan Cement Association (JCA) [22] and the Ministry of Transportation and Communications of China (MOT) [22]. The relationships are presented in Equations (16) and (17).

(16) $$f_{cu,m}=7.82\;f_{t,m}-15.56$$

(17) $$f_{t,m}=0.453\;f_{cu,m}^{0.713}$$

Substituting *f_c_* = 0.76 *f_cu_* into Equations (16) and (17) yields the following:

(18) $$f_{c,m}=10.29\;f_{t,m}-20.47$$

(19) $$f_{t,m}=0.372\;f_{c,m}^{0.713}$$

**Figure 14:**
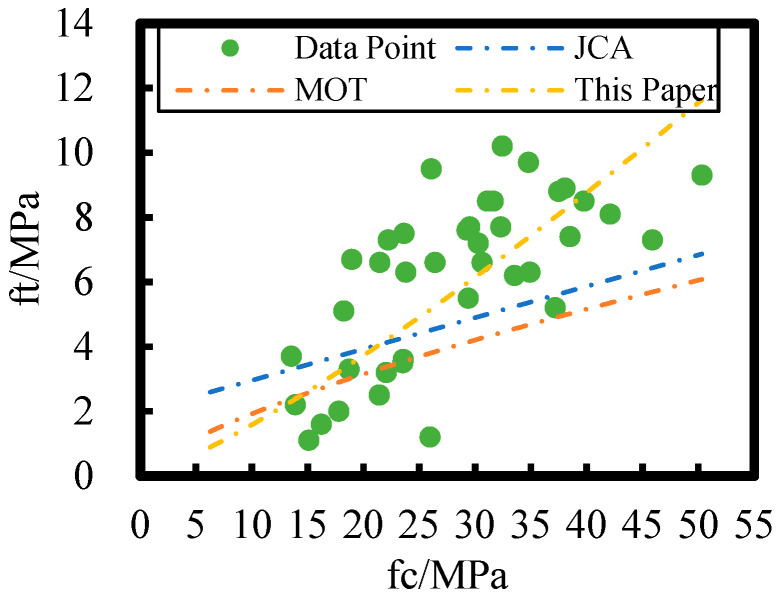
Comparison of different flexural strength–axial compressive strength models with the flexural strength–axial compressive strength model in this paper.

The relationship between flexural and axial compressive strengths of HPC after 8 years of on-site exposure in the Salt Lake region is plotted against different models in Figure 14; the loss rate of axial compressive strength of HPC at the Salt Lake exposure site is higher than that of flexural strength relative to non-corroded concrete. Therefore, the JCA and MOT concrete flexural strength–axial compressive strength models are not applicable to HPC exposed to the Salt Lake environment. However, there remains an overall power function relationship between the flexural and axial compressive strengths of HPC in this study.

**Figure 15:**
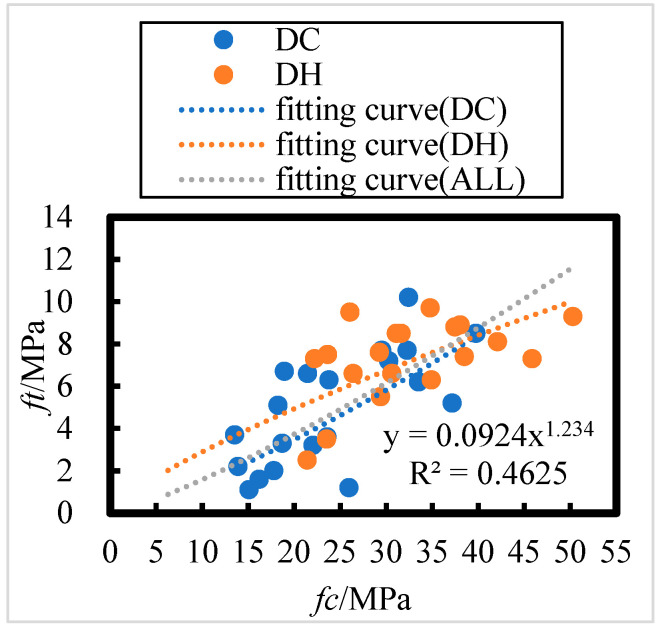
Relationship between HPC flexural strength and axial compressive strength in dry cold and dry heat initial incubation environments under 8 years of field exposure in the Salt Lake region.

The relationship between HPC flexural and axial compressive strengths after 8 years of field exposure to lower DC and DH initial environments in the Salt Lake region is shown in Figure 15; the relationship between flexural strength (*f_t_*) and axial compressive strength (*f_c_*) can be summarized as follows:

(20) $$f_{t}=0.4922\;f_{c}^{0.7694}\;\;\;\;\mathrm{DH},\;R\;=\;0.55$$

(21) $$f_{t}=0.0756\;f_{c}^{1.2771}\;\;\;\;\mathrm{DC},\;R\;=\;0.63$$

(22) $$f_{t}=0.0924\;f_{c}^{1.234}\;\;\;\;\mathrm{ALL},\;R\;=\;0.69$$

The number of HPC specimens with initial curing environments of DH and DC are 19 and 21, respectively. Referring to the Table 8, the critical correlation coefficients at a significance level of 0.01 for n = 19 and n = 21 are 0.549 and 0.526, respectively. For all HPC samples (n = 40), the critical correlation coefficient at a significance level of 0.001 is 0.49. All correlation coefficients passed the correlation coefficient test, indicating a highly significant power function relationship between the flexural and axial compressive strengths of concrete after 8 years of exposure in the Salt Lake region.

As shown in Figure 15, the flexural strength of concrete after 8 years of exposure to DH and DC initial curing environments in the Salt Lake region follows a similar trend to that of axial compressive strength. This suggests that the DH and DC initial curing environments have minimal effect on the relationship between flexural and axial compressive strengths. Flexural strength is positively correlated with axial compressive strength, and there exists a power function relationship between the two.

##### Relationship Between Splitting Tensile and Axial Compressive Strengths

Various associations and departments domestically and internationally have established various models for the relationship between splitting tensile and compressive strengths of concrete, such as the American Concrete Institute (ACI) [23], the European Concrete Institute and the International Prestressed Concrete Association (CEB-FIP) [24], and the State Administration of Standardization of China (SAC) [21].

(23) $$f_{cu,m}=21\sim 83\;\mathrm{MPa}:\;f_{sp,m}=0.59\;f_{cu,m}^{0.5}$$

(24) $$f_{sp,m}=0.301\;f_{cu,m}^{0.67}$$

(25) $$f_{sp,m}=0.19\;f_{cu,m}^{0.75}$$

Substituting *f_c_* = 0.76 *f_cu_* into Equations (23)–(25) yields

(26) $$f_{c,m}=16\sim 63\;\mathrm{MPa}:\;f_{sp,m}=0.51\;f_{c,m}^{0.5}$$

(27) $$f_{sp,m}=0.25\;f_{c,m}^{0.67}$$

(28) $$f_{sp,m}=0.15\;f_{c,m}^{0.75}$$

**Figure 16:**
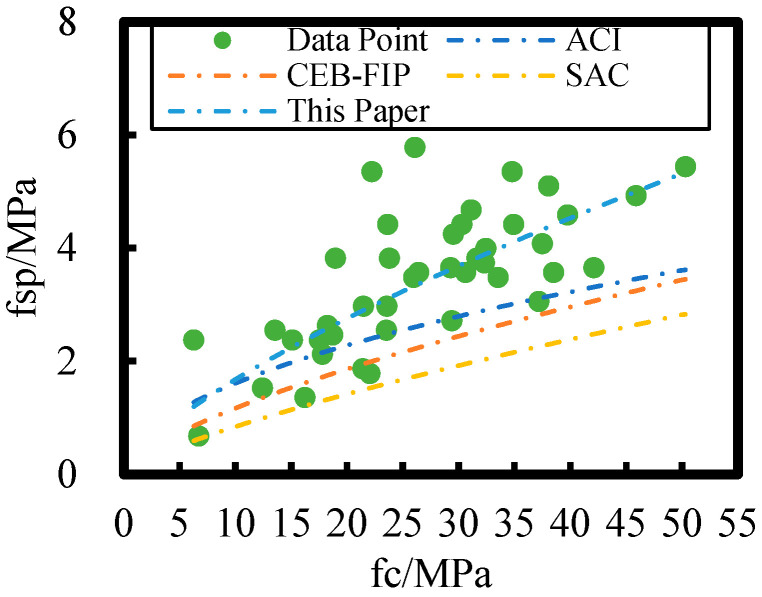
Comparison of different splitting tensile strength–axial compressive strength models with the splitting tensile strength–axial compressive strength model in this paper.

The relationship between split tensile and axial compressive strengths of HPC after 8 years of on-site exposure in the Salt Lake region is plotted for different models in Figure 16; relative to the non-corroded concrete, the rate of loss of axial compressive strength of HPC in the Salt Lake exposure site is higher than that of the split tensile strength. Therefore, the split tensile strength–axial compressive strength models of ACI, CEB-FIP, and SAC are not applicable for flexural strength–axial compressive strength modeling of HPC at the salt lake exposure site. In this study, the HPC splitting tensile and axial compressive strengths have a power function relationship.

**Figure 17:**
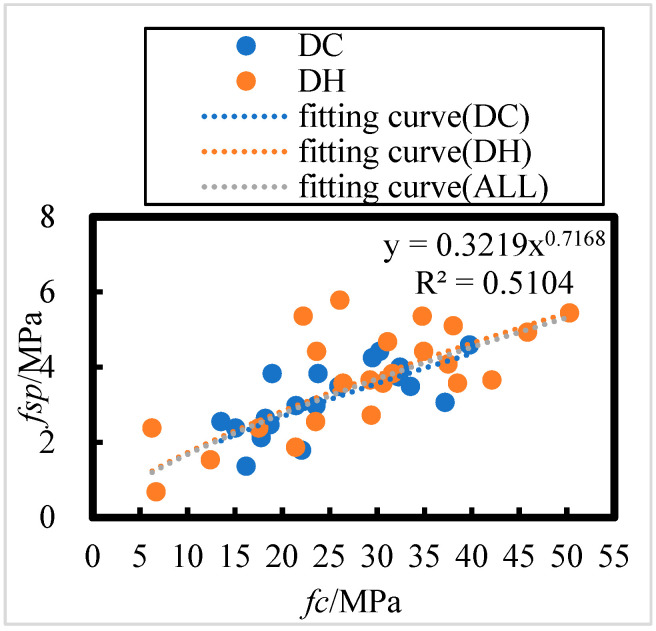
Relationship between HPC splitting tensile strength and axial compressive strength in dry cold and dry heat initial curing environments under 8 years of on-site exposure in the Salt Lake region.

The relationship between splitting tensile and axial compressive strengths of DC- and DH-exposed HPC after 8 years of field exposure in the Salt Lake region is shown in Figure 17; the relationship between split tensile strength (*f_sp_*) and axial compressive strength (*f_c_*) can be summarized as follows:

(29) $$f_{sp}=0.3329\;f_{c}^{0.7139}\;\;\mathrm{DH},\;R\;=\;0.77$$

(30) $$f_{sp}=0.3294\;f_{c}^{0.7003}\;\;\mathrm{DC},\;R\;=\;0.69$$

(31) $$f_{sp}=0.3219\;f_{c}^{0.7168}\;\;\mathrm{ALL},\;R\;=\;0.75$$

The number of HPC specimens with initial curing environments of DH and DC is 23 and 20, respectively. According to Table 8, the critical correlation coefficients at a 0.01 level of significance for n = 23 and n = 20 are 0.505 and 0.537, respectively. For all the HPC sample specimens (n = 43), the critical correlation coefficient at a 0.001 level of significance is 0.474. All correlation coefficients passed the correlation coefficient test, indicating a highly significant power function relationship between concrete splitting tensile and axial compressive strengths after 8 years of exposure in the Salt Lake region.

As shown in Figure 17, the concrete splitting tensile strength after 8 years of exposure to DH and DC priming environments in the Salt Lake region follows a similar trend to axial compressive strength. This suggests that the DH and DC priming environments have a minimal effect on the relationship between concrete splitting tensile and compressive strengths under these conditions. The splitting tensile strength is positively correlated with compressive strength, and a power function relationship exists between the two.

### 3.3. Relationship Between Strength and Corrosion Damage of HPC After 8 Years of Exposure in the Salt Lake Region

#### 3.3.1. Corrosion Damage with Different Mixing Ratios

The relative dynamic modulus (*E_rd_*) of concrete and internal corrosion damage are calculated according to Equations (4) and (5), and the results are listed in Table 9 and Table 10, respectively. For cases where corrosion damage, *D*, is negative, according to Equation (5), it indicates that no corrosion damage has occurred in the concrete. This phenomenon suggests that the internal structure has become denser owing to the volcanic ash reaction of mineral admixtures and the filling of corrosion products within a certain depth range of the surface layer. From Table 9 and Table 10, it can be seen that in 27.5% of the HPC specimens, corrosion damage is 0; in 70% of the HPC specimens, corrosion damage is between 0 and 0.3; and in only one specimen, corrosion damage is approximately 0.4. This indicates that the HPC prepared herein has good performance in terms of internal corrosion resistance.

#### 3.3.2. Relationship Between Splitting Tensile Strength and Corrosion Damage of HPC

**Figure 18:**
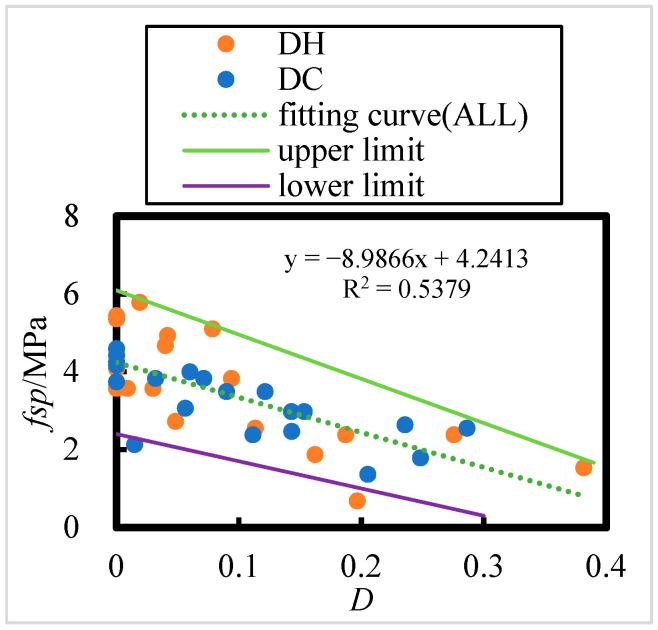
Split tensile strength of DC- and DH-exposed HPC versus corrosion damage, D.

The relationship between the splitting tensile strength of HPC after 8 years of exposure in the Salt Lake region and the corrosion damage (*D*) is shown in Figure 18; there exists a linear relationship between the splitting tensile strength and the corrosion damage (*D*).

(32) $$f_{\mathrm{sp}}=-9.7786D+4.4304\;\;\;\;\;\mathrm{DH},\;R\;=\;0.74$$

(33) $$f_{\mathrm{sp}}=-7.3381D+3.9277\;\;\;\;\;\mathrm{DC},\;R\;=\;0.73$$

(34) $$f_{\mathrm{sp}}=-8.9866D+4.2413\;\;\;\;\;\mathrm{ALL},\;R\;=\;0.73$$

The number of HPC specimens with initial curing environments of DH and DC is 23 and 21, respectively. According to the Table 8, the critical correlation coefficients at a significance level of 0.001 for n = 23 and n = 21 are 0.618 and 0. 64, respectively. For all HPC samples are (n = 44), the critical correlation coefficient at the 0.001 significance level is 0.469. All correlation coefficients passed the correlation coefficient test.

This indicates that the existence of a significant linear relationship between corrosion damage and splitting tensile strength under DC and DH curing conditions and a highly significant linear relationship with overall splitting tensile strength. The corrosion damage is inversely proportional to the splitting tensile strength, indicating that as corrosion damage increases, the splitting tensile strength decreases after 8 years of exposure in the Salt Lake region. Furthermore, the corrosion damage caused by exposure in the Salt Lake region has a noticeable effect on the concrete’s overall strength.

### 3.4. Relationship Between Corrosion Resistance Coefficient, Relative Dynamic Elastic Modulus, and Mix Ratio of HPC

#### 3.4.1. Relationship Between Concrete Corrosion Resistance Factor and Mix Ratio

The calculation of the concrete corrosion resistance coefficient (*K*) is as per Equation (6); the results are listed in Table 11 and Table 12.

**Figure 19:**
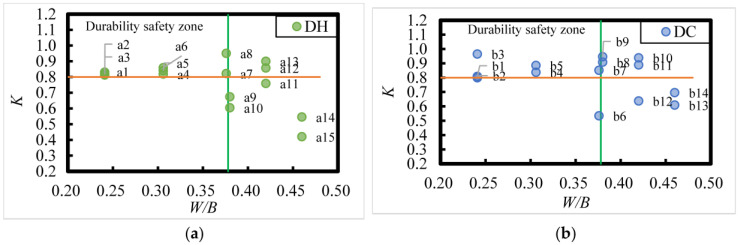
Relationship between the corrosion resistance coefficients (K) and mix ratio of DC- and DH-exposed HPC under 8 years of exposure in the Salt Lake region. (**a**) DH; (**b**) DC. Register [n (*W*/*B*; FA content)]: a1 (0.241; 15%), a2 (0.241; 25%), a3 (0.241; 35%), a4 (0.306; 15%), a5 (0.306; 25%), a6 (0.306; 35%), a7 (0.376; 15%), a8 (0.376;25%), a9 (0.38; 23%), a10 (0.38; 43%), a11 (0.42; 23%), a12 (0.42; 33%), a13 (0.42; 43%), a14 (0.46; 33%), a15 (0.46; 43%); b1 (0.241; 15%), b2 (0.241; 25%), b3 (0.241; 35%), b4 (0.306; 15%), b5 (0.306; 25%), b6 (0.376; 15%), b7 (0.376; 25%), b8 (0.38; 23%), b9 (0.38; 33%), b10 (0.42; 23%), b11 (0.42; 33%), b12 (0.42; 43%), b13 (0.46; 23%), b14 (0.46; 43%).

The corrosion resistance coefficient, *K*, of DC- and DH-exposed HPC after 8 years of exposure in the Salt Lake area is shown in Figure 19. A corrosion resistance coefficient of concrete *K* ≥ 0.8 indicates better corrosion resistance. From Figure 19, it is evident that the corrosion resistance of HPC after 8 years of exposure in the Salt Lake area is related to the *W*/*B*, and the larger the *W*/*B*, the worse the corrosion resistance. The corrosion resistance of HPC is better when *W*/*B* is less than 0.24 and worse when *W*/*B* is larger than 0.38. According to the distribution pattern of experimental data points measured on concrete specimens in the upper and lower regions of the corrosion resistance coefficient of 0.80, it is evident that the corrosion resistance coefficient is greater than 0.80 for HPC with *W*/*B* of 0.241 and 0.306, with FA admixtures ranging from 15% to 35%, and with SF of 10%. When *W*/*B* is 0.38, 0.42, and 0.46, respectively, 27%, 33%, and 100% of the HPC specimens had corrosion resistance coefficients lower than 0.80. In addition, it can be seen that for *W*/*B* larger than 0.38, the HPC with better corrosion resistance had FA content greater than 25%, indicating that increasing the FA content can improve the corrosion resistance of the HPC to some extent.

#### 3.4.2. Relative Dynamic Elastic Modulus of DC- and DH-Exposed HPC as a Function of Fit Ratio

**Figure 20:**
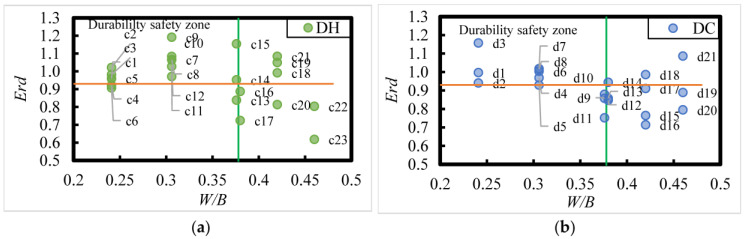
Relative dynamic elastic modulus (*E_rd_*) of Dry Cold and Dry Heat HPC versus fit ratio under 8 years of exposure in the Salt Lake region. (**a**) DH; (**b**) DC. Register [n (*W*/*B*; FA content)]:c1 and c2 (0.241; 15%), c3 and c4 (0.241; 25%), c5 and c6 (0.241; 35%), c7 and c8 (0.306; 15%), c9 and c10 (0.306; 25%), c11 and c12 (0.306; 35%), c13 and c14 (0.376; 15%), c15 (0.376; 25%), c16 (0.38; 23%), c17 (0.38; 43%), c18 (0.42; 23%), c19 and c20 (0.42; 33%), c21 (0.42; 43%), c22 (0.46; 33%), c23 (0.46; 43%); d1 (0.241; 15%), d2 (0.241; 25%), d3 (0.241; 35%), d4 and d5 and d6 (0.306; 15%), d7 and d8 (0.306; 25%), d9 and d10 (0.376; 15%), d11 (0.376; 25%), d12 and d13 (0.38; 23%), d14 (0.38; 33%), d15 and d16 (0.42; 23%), d17 (0.42; 33%), d18 (0.42; 43%), d19 and d20 (0.46; 23%), d21 (0.46; 43%).

The relative dynamic modulus (*E_rd_*) of DC- and DH-exposed HPC after 8 years of exposure in the Salt Lake region is shown in Figure 20; it can be observed that an Erd ≥ 0.93 indicates better durability of HPC after 8 years of exposure to the saline soil environment in the Salt Lake region. Additionally, the durability of HPC in this environment is closely related to the *W*/*B*. When *W*/*B* is between 0.24 and 0.38, the durability of HPC with 15% to 35% FA and 10% SF admixture was better. However, when *W*/*B* is larger than 0.38, the durability of HPC decreased.

According to the distribution pattern of experimental data points measured in the concrete specimens, when the relative dynamic modulus of elasticity exceeds 0.93, the durability of HPC improves. In contrast, when *W*/*B* is 0.241, 0.38, 0.42, and 0.46, 25%, 73%, 50%, and 80% of the HPC specimens exhibit poorer durability. Furthermore, it was found that for *W*/*B* larger than 0.38, increasing the FA content to more than 25% improved the durability of HPC, suggesting that higher FA content can enhance the durability of HPC under certain conditions.

#### 3.4.3. Corrosion Resistance Coefficients of DC- and DH-Exposed HPC in Relation to 28 d Standardized Curing Strength

**Figure 21:**
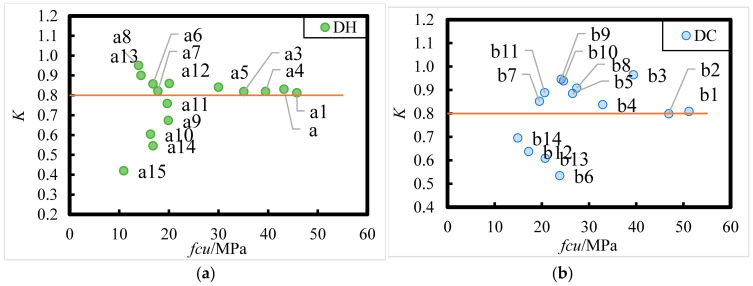
Corrosion resistance coefficient (*K*) versus 28 d standard curing strength for Dry Cold and Dry Heat HPC under 8 years of exposure in the Salt Lake region. (**a**) DH; (**b**) DC. Register [n (*W*/*B*; FA content)]:a1 (0.241; 15%), a2 (0.241; 25%), a3 (0.241; 35%), a4 (0.306; 15%), a5 (0.306; 25%), a6 (0.306; 35%), a7 (0.376; 15%), a8 (0.376; 25%), a9 (0.38; 23%), a10 (0.38; 43%), a11 (0.42; 23%), a12 (0.42; 33%), a13 (0.42; 43%), a14 (0.46; 33%), a15 (0.46; 43%); b1 (0.241; 15%), b2 (0.241; 25%), b3 (0.241; 35%), b4 (0.306; 15%), b5 (0.306; 25%), b6 (0.376; 15%), b7 (0.376; 25%), b8 (0.38; 23%), b9 (0.38; 33%), b10 (0.42; 23%), b11 (0.42; 33%), b12 (0.42; 43%), b13 (0.46; 23%), b14 (0.46; 43%).

The relationship between the corrosion resistance coefficient (K) of HPC and the 28 d standard curing strength after 8 years of exposure in the Salt Lake region is shown in Figure 21. A corrosion resistance coefficient (K ≥ 0.80) indicates that the concrete has better corrosion resistance. From Figure 21, it can be observed that the corrosion resistance of HPC after 8 years of exposure in the Salt Lake region is related to the standard 28 d hardening strength. The corrosion resistance of HPC is poor when the 28 d standard hardening strength is less than 25 MPa, and better when the 28 d standard hardening strength is greater than or equal to 25 MPa.

According to the distribution of experimental data points measured in the concrete specimens, it was found that for HPC specimens with water–binder ratios (*W*/*B*) of 0.241 and 0.306, FA content between 15% and 35%, and SF content of 10%, the corrosion resistance coefficients are greater than 0.80. In contrast, when *W*/*B* is 0.38, 0.42, and 0.46, 37.5%, 42.9%, and 100% of HPC specimens, respectively, have corrosion resistance coefficients lower than 0.80. Furthermore, it was observed that for *W*/*B* larger than 0.38, HPC with better corrosion resistance had FA content greater than 25%, indicating that increasing the FA content can improve the corrosion resistance of HPC to a certain extent.

### 3.5. Metrics for Judging the Durability of HPC After 8 Years of Exposure in the Salt Lake Region—Relative Dynamic Elastic Modulus Versus Corrosion Resistance Factor

**Figure 22:**
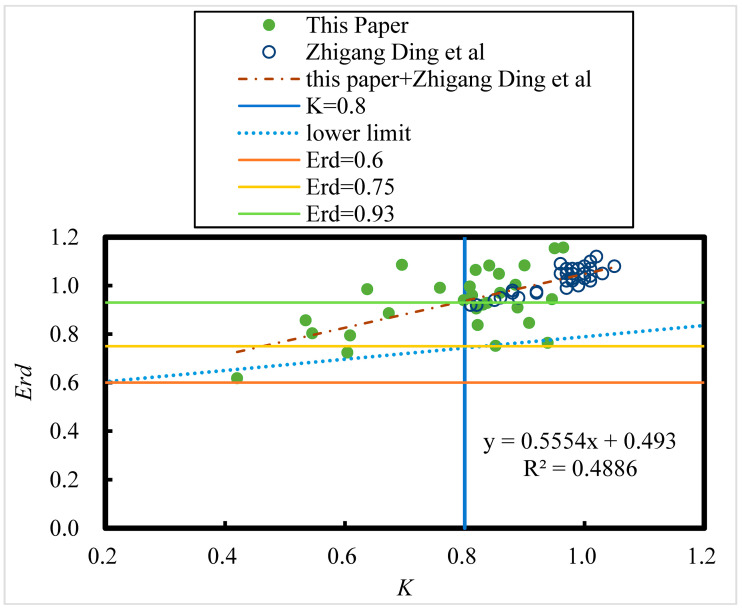
Relative dynamic elastic modulus (*E_rd_*) versus corrosion resistance coefficient (K) of HPC under 8 years of exposure in the Salt Lake region [18].

The relationship between the relative kinetic elastic modulus (*E_rd_*) of HPC and the corrosion resistance coefficient (*K*) after 8 years of exposure in the Salt Lake region is shown in Figure 22.

(35) $$E_{\mathrm{rd}}=0.5554K+0.493\;\;\;R\;=\;0.70$$

The number of samples of all HPC specimens is 64, and from the Table 8, the critical correlation coefficient for a significance level of 0.001 at n = 64 is 0.396; the correlation coefficient passed the correlation coefficient test. Therefore, the relative dynamic elastic modulus *E_rd_* of HPC has a highly significant linear relationship with the corrosion resistance coefficient K after 8 years of exposure in the salt lake area. According to the relative kinetic elastic modulus regulations of concrete durability experiments, concrete durability damage is considered to have occurred when the relative kinetic elastic modulus reaches 0.60. However, as shown in Figure 21, for HPC exposed to Salt Lake for 8 years, the corrosion resistance coefficient is only 0.2 when the relative kinetic elastic modulus reaches 0.60. The strength of the concrete decreases by as much as 80% at this time. It can be seen that following the relative dynamic elastic modulus up to 0.60 as a control index for durability damage for field corroded concrete in the Salt Lake area with high soluble salt content is not feasible. From Equation (35), it is found that the axial compressive strength corrosion resistance factor corresponding to the HPC relative kinetic elastic modulus up to 0.93 is about 0.8 (i.e., the strength is lost by 20%); from the lower limit curve, it corresponds to the axial compressive strength corrosion resistance factor corresponding to the HPC relative kinetic elastic modulus up to 0.73, which is about 0.8. Therefore, the average sign of damage for the concrete with the relative kinetic elastic modulus is 0.93, and the lowest breakage sign is 0.73 instead of 0.60.

### 3.6. Corrosion Products and Micro-Mechanisms

**Figure 23:**
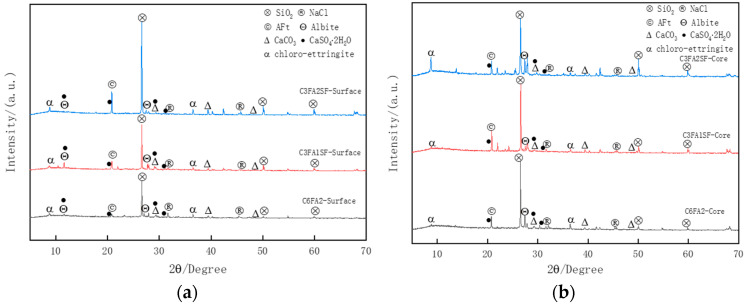
XRD spectrum of C3FA1SF, C3FA2SF, and C6FA2 exposed in salt lake site for 8 years. (**a**) Surface. (**b**) Core.

To investigate the corrosion damage of HPC, this paper conducted an XRD microscopic analysis of the mortar within 3 mm from the surface of concrete. Figure 23. shows the XRD spectrum of C3FA1SF, C3FA2SF, and C6FA2 exposed in a salt lake site for 8 years, where quartz (SiO_2_) and albite (Na_2_O·Al_2_O_3_·6SiO_2_) are impurities from the raw materials in the concrete sample. The corrosion products of brine include physical corrosion products, such as NaCl, and chemical corrosion products like ettringite (AFt, 3CaO·Al_2_O_3_·3CaSO_4_·31H_2_O), chloro-ettringite (3CaO·Al_2_O_3_·3CaCl_2_·30H_2_O), and calcium sulfate dihydrate (CaSO_4_·2H_2_O). Meanwhile, a certain portion of AFt also originates from the secondary hydration products of mineral admixtures. Calcium carbonate (CaCO_3_) mainly belongs to the carbonation products of Ca(OH)_2_ (CH). To further investigate the internal corrosion damage of HPC, this paper conducted SEM analysis of the microstructure of mortar samples on the surface and core of concrete.

**Figure 24:**
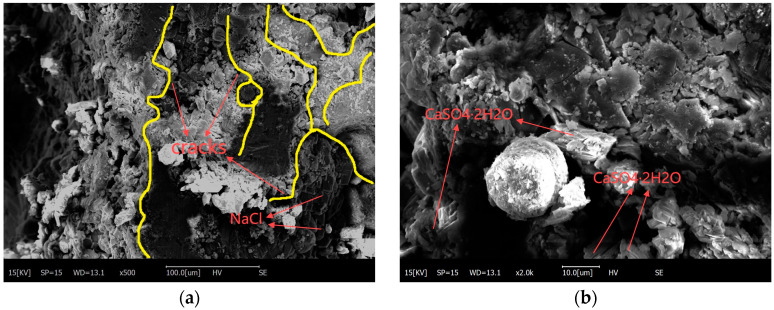

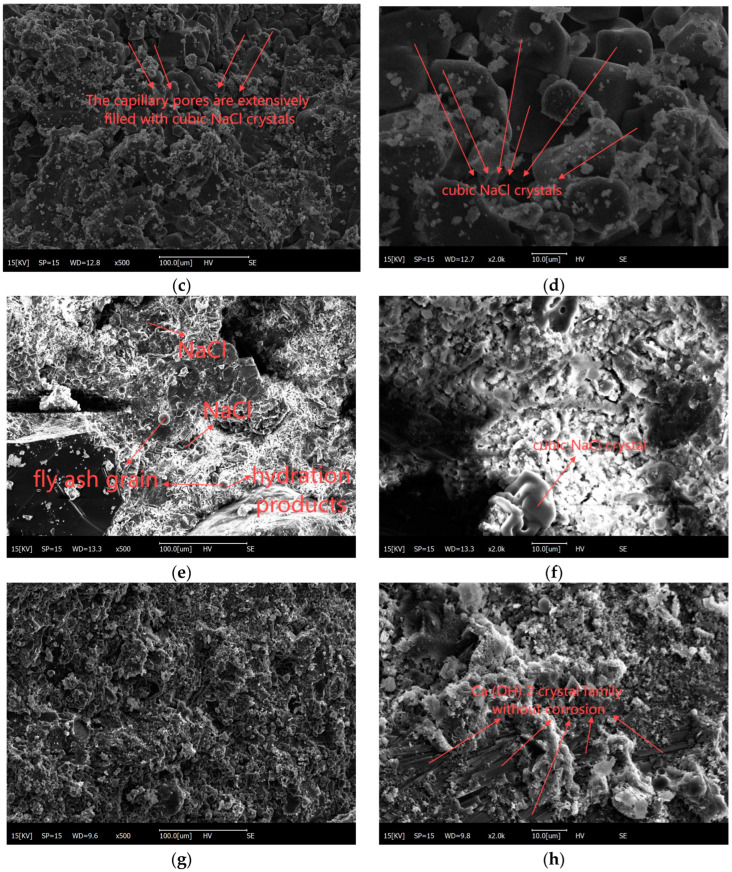
SEM image of C3FA2SF and C6FA2 exposed in salt lake site for 8 years. (**a**) C6FA2-Surface-500×. (**b**) C6FA2-Surface-2000×. (**c**) C6FA2-Core-500×. (**d**) C6FA2-Core-2000×. (**e**) C3FA2SF-Surface-500×. (**f**) C3FA2SF-Surface-2000×. (**g**) C3FA2SF-Core-500×. (**h**) C3FA2SF-Core-2000×.

Figure 24 illustrates the SEM images of the mortar layer of HPC with the mix proportion of C3FA2SF (K = 0.95) and C6FA2 (K = 0.42) after being exposed in salt lake site for 8 years. From Figure 24a–d, it can be observed that the surface and core mortar of the low-corrosion-resistance HPC was severely corroded, with corrosion products distributed throughout both the surface and core mortar, large pores and cracks in the mortar, and fly ash particles that were difficult to locate. From Figure 24e,f, it can be observed that the microstructure of the surface mortar of the high-corrosion-resistance HPC was relatively intact. Slight cracks appeared in the mortar and extended along the pores, but these cracks did not damage the overall structure; the pores were filled with sodium chloride and the fly ash was partially hydrated. From Figure 24g,h, it can be observed that the core mortar microstructure of HPC with high corrosion resistance was relatively intact; slight pores appeared in the mortar, and there was a lot of sodium chloride on the surface of the mortar as well as in the pores in the mortar, which did not participate in the chemical reaction.

## 4. Conclusions

In this paper, mechanical properties, corrosion damage evolution laws, and judgment indicators of durability damage of HPC under a long-term (2920 d) brine corrosion environment were studied, and the main conclusions were drawn as follows:

(1) There is a highly significant negative linear correlation between the water–binder ratio and the compressive, flexural, and splitting tensile strengths of HPC after 8 years of exposure in the Salt Lake region. As the water–binder ratio increases, the strength of HPC decreases, with higher water–binder ratios resulting in a more pronounced loss of strength. FA content has a notable effect on the compressive, flexural, and splitting tensile strengths of HPC after 8 years of exposure in the Salt Lake region. This effect is more prominent in HPC with a design strength grade of C20 to C40, where higher FA content leads to higher strength. However, the effect is less significant in HPC with a design strength grade of C30 to C70;
(2) The corrosion resistance of HPC after 8 years of exposure in the Salt Lake region is related to the 28 d standard hardening strength. The corrosion resistance of HPC was poor when the 28 d standard yield strength was less than 25 MPa, and it was better when the 28 d standard yield strength was greater than or equal to 25 MPa;
(3) The durability of HPC after 8 years of exposure in the salt lake area is related to the water–binder ratio and mineral admixture. When *W*/*B* is between 0.24 and 0.38, HPC with 15%–35% FA and 10% SF had better corrosion resistance; when *W*/*B* is larger than 0.38, HPC with 23% FA had worse corrosion resistance; for HPC with *W*/*B* larger than 0.38, HPC had better corrosion resistance when FA doping was greater than 25%;
(4) For HPC subjected to the strong saline soil environment in the Qarhan Salt Lake area of Qinghai, after 8 years of corrosion, the durability damage markers can be selected from the relative dynamic elastic modulus and corrosion resistance coefficient. When the relative dynamic elastic modulus *E_rd_* is lower than 0.73–0.93 and the corrosion resistance coefficient K is lower than 0.8, it can be assumed that the corrosion damage of the concrete specimen has already occurred.
(5) In the saline soil environment of Qarhan Salt Lake in Qinghai, the corrosion products of brine on concrete include physical corrosion products, such as NaCl, and chemical corrosion products like ettringite, chloro-ettringite, and calcium sulfate dihydrate. The surface and core of concrete with poor corrosion resistance suffer from severe physical and chemical corrosion, resulting in a fragmented structure; concretes with strong corrosion resistance suffer with only surface corrosion and have an intact interior without obvious micro cracks.

As HPC was only exposed for 8 years at the salt lake site, it was not possible to record changes in its strength over its lifetime, which may be a limitation of this study. In the future, it may be possible to extend the exposure of HPC at the salt lake site to 15, 30, or even more years to better understand the evolution of its strength over time and to predict its service life in such environments.

## Figures and Tables

**Table 1:** Physical and Mechanical Properties of Cement.

Fineness /%	Specific Surface Area /m^2^·kg^−1^	Coagulation Time		Flexural Strength/MPa		Compressive Strength/MPa	
0.8	348	Initial set	Final set	3 d	28 d	3 d	28 d
		2:25	3:40	5.5	7.6	21.6	48.7

**Table 2:** Chemical composition of cementitious materials/%.

Materials	SiO_2_	Al_2_O_3_	CaO	MgO	SO_3_	Fe_2_O_3_	Na_2_O	TiO_2_	K_2_O	MnO_2_
P.O 42.5 cement	21.62	9.59	59.98	1.94	1.65	3.69	0.60	0.04	0.82	0.07
Fly ash	45	32	8.54	0.99	2.5	7.43	1.32	0.42	1.51	0.20
Silica fume	94.82	0.36	0.11	1.22	-	0.34	0.53	1.07	1.46	0.09

**Table 3:** C20~C40 Concrete Mix Proportions.

Number	*W*/*B*	Crushed Stone /kg·m^−3^	Sand /kg·m^−3^	Water /kg·m^−3^	Cement /kg·m^−3^	FA/%	HAJ-2/%
C4FA1	0.38	1075	778	160	323	23	1
C4FA2	0.38	1075	778	160	281	33	1
C4FA3	0.38	1075	778	160	239	43	1
C5FA1	0.42	1075	778	176	323	23	1
C5FA2	0.42	1075	778	176	281	33	1
C5FA3	0.42	1075	778	176	239	43	1
C6FA1	0.46	1075	778	193	323	23	1
C6FA2	0.46	1075	778	193	281	33	1
C6FA3	0.46	1075	778	193	239	43	1

**Table 4:** C30~C70 Concrete Mix Proportions.

Number	*W*/*B*	Crushed Stone /kg·m^−3^	Sand /kg·m^−3^	Water /kg·m^−3^	Cement /kg·m^−3^	FA/%	SF/%	FDN/%
C1FA1SF	0.241	957	752	131	408	15	10	0.85
C1FA2SF	0.241	957	752	131	354	25	10	0.85
C1FA3SF	0.241	957	752	131	299	35	10	0.85
C2FA1SF	0.306	957	752	167	408	15	10	0.85
C2FA2SF	0.306	957	752	167	354	25	10	0.85
C2FA3SF	0.306	957	752	167	299	35	10	0.85
C3FA1SF	0.376	957	752	204	408	15	10	0.85
C3FA2SF	0.376	957	752	204	354	25	10	0.85
C3FA3SF	0.376	957	752	204	299	35	10	0.85

**Table 5:** C20~C40 Concrete Compressive Strength at 28 Days/MPa.

Number	Standard Curing	Dry Heat Curing	Dry Cold Curing
C4FA1	44.6	19.9	27.4
C4FA2	39.5	20.4	24.1
C4FA3	32.2	16.3	21.7
C5FA1	43.5	19.7	24.6
C5FA2	34.7	16.8	20.6
C5FA3	25.1	14.4	17.2
C6FA1	32.4	20.0	20.7
C6FA2	26.1	16.8	17.1
C6FA3	21.6	10.9	14.9

**Table 6:** C30~C70 Concrete Compressive Strength at 28 Days/MPa.

Number	Standard Curing	Dry Heat Curing	Dry Cold Curing
C1FA1SF	74.9	45.8	51.2
C1FA2SF	74.6	43.2	46.9
C1FA3SF	67.7	39.5	39.4
C2FA1SF	63.7	35.1	32.9
C2FA2SF	60.6	30.0	26.5
C2FA3SF	48.1	20.1	24.3
C3FA1SF	41.8	17.8	23.8
C3FA2SF	32.1	13.9	19.5
C3FA3SF	33.0	14.0	18.0

**Table 7:** Chemical composition of soluble salts in the surface soil of Qarhan Salt Lake in Qinghai/% [17].

Sample	K^+^	Na^+^	Mg^2+^	Ca^2+^	Cl^−^	SO_4_^2−^	CO_3_^2−^	Total
Potassic Fertilizer Plant in Qinghai Salt Lake	0.64	1.85	1.99	4.81	12.86	0.48	3.84	26.47
185# steel tower of Ge-Cha transmission line	0.08	10.04	0.50	3.66	17.88	0.70	4.31	37.17
At the 618 km milepost of Qinghai–Xizang Road	0.25	10.40	0.64	4.63	10.42	9.70	7.40	43.66

**Table 8:** Correlation coefficient (R) significance test table (partial).

Number of Samples	0.05	0.02	0.01	0.005	0.002	0.001
19	0.433	0.503	0.549	0.589	0.635	0.665
20	0.423	0.492	0.537	0.576	0.622	0.652
21	0.413	0.482	0.526	0.565	0.610	0.640
23	0.396	0.462	0.505	0.543	0.588	0.618
64	0.242	0.286	0.315	0.342	0.374	0.396

Register: 0.05 indicates significant level; 0.001 indicates highly significant level.

**Table 9:** Corrosion damage of HPC in the dry and hot initial curing environment in the Salt Lake area exposed for 8 years.

Number	*E_rd_*	*D*	Number	*E_rd_*	*D*
C1FA1SF-1	0.96	0.04	C3FA1SF-1	0.84	0.16
C1FA1SF-2	1.02	0	C3FA1SF-2	0.95	0.05
C2FA2SF-1	0.98	0.02	C3FA2SF	1.15	0
C1FA1SF-2	0.92	0.08	C4FA1	0.89	0.11
C1FA3SF-1	0.96	0.04	C4FA3	0.72	0.28
C1FA3SF-2	0.91	0.09	C5FA1	0.99	0.01
C2FA1SF-1	1.07	0	C5FA2-1	1.05	0
C2FA1SF-2	1.06	0	C5FA2-2	0.81	0.19
C2FA2SF-1	1.19	0	C5FA3	1.08	0
C2FA2SF-2	1.08	0	C6FA2	0.80	0.20
C2FA3SF-1	0.97	0.03	C6FA3	0.62	0.38
C2FA3SF-2	1.03	0			

**Table 10:** Corrosion damage of HPC in the dry and cold initial curing environment in the Salt Lake area exposed for 8 years.

Number	*E_rd_*	*D*	Number	*E_rd_*	*D*
C1FA1SF	1.00	0.00	C4FA1-1	0.85	0.15
C1FA2SF	0.94	0.06	C4FA1-2	0.86	0.14
C1FA3SF	1.16	0	C4FA2	0.94	0.06
C2FA1SF-1	0.97	0.03	C5FA1-1	0.76	0.24
C2FA1SF-2	0.93	0.07	C5FA1-2	0.71	0.29
C2FA2SF-1	1.00	0	C5FA2	0.91	0.09
C2FA2SF-2	1.02	0	C5FA3	0.99	0.01
C2FA2SF-3	1.02	0	C6FA1-1	0.89	0.11
C3FA1SF-1	0.86	0.14	C6FA1-2	0.79	0.21
C3FA1SF-2	0.88	0.12	C6FA3	1.09	0
C3FA2SF	0.75	0.25			

**Table 11:** Corrosion resistance coefficients for HPC in dry heat initial curing environments under 8 years of exposure in the Salt Lake region.

Number	*K*	Number	*K*
C1FA1SF	0.81	C3FA2SF	0.95
C1FA2SF	0.83	C4FA1	0.67
C1FA3SF	0.82	C4FA3	0.60
C2FA1SF	0.82	C5FA2	0.86
C2FA2SF	0.84	C5FA3	0.90
C2FA3SF	0.86	C6FA2	0.55
C3FA1SF	0.82	C6FA3	0.42

**Table 12:** Corrosion resistance coefficients for HPC in dry cold initial curing environments under 8 years of exposure in the Salt Lake region.

Number	*K*	Number	*K*
C1FA1SF	0.81	C4FA1	0.91
C1FA2SF	0.80	C4FA2	0.95
C1FA3SF	0.96	C5FA1	0.94
C2FA1SF	0.84	C5FA2	0.89
C2FA2SF	0.88	C5FA3	0.64
C3FA1SF	0.53	C6FA1	0.61
C3FA2SF	0.85	C6FA3	0.70

## Data Availability

The original contributions presented in the study are included in the article, further inquiries can be directed to the corresponding authors.
